# Biologics and Pediatric Generalized Pustular Psoriasis: An Emerging Therapeutic Trend

**DOI:** 10.7759/cureus.652

**Published:** 2016-06-22

**Authors:** Sami K Saikaly, Monica Mattes

**Affiliations:** 1 College of Medicine, University of Central Florida College of Medicine

**Keywords:** pediatric, psoriasis, generalized pustular psoriasis, biologics, etanercept, adalimumab, infliximab, gpp

## Abstract

Generalized pustular psoriasis (GPP) is a rare form of childhood psoriasis, often requiring systemic therapy, which is challenging as there is a paucity of randomized controlled trials and standardized guidelines. Biologic agents have been used in adults and in pediatric plaque psoriasis, but evidence regarding their efficacy in pediatric GPP has slowly become available. The objective of this study is to summarize and compare the efficacy and safety of biologic agents, such as etanercept, infliximab, and adalimumab, in the treatment of pediatric GPP. A PubMed literature review was conducted and 12 studies met the inclusion criteria for analysis. After reviewing the efficacy of these drugs in pediatric GPP patients and their safety in the use of other pediatric conditions, etanercept was identified as a possible first-line biologic agent for pediatric psoriasis, including GPP, followed by infliximab and adalimumab. In conclusion, several case reports have documented the successful use of biologic agents in refractory cases of pediatric GPP, but clinical trials are needed to gain a better understanding of the efficacy and side effect profile in this population.

## Introduction and background

Psoriasis, affecting 2 to 4% of the world’s population [1-2], is a chronic T-cell-mediated inflammatory disease marked by keratinocyte hyperproliferation [2]. Many subtypes of psoriasis have been described, including generalized pustular psoriasis (GPP) by von Zumbusch in 1910. It is widely reported that the first signs of psoriasis appear prior to the age of 20 in approximately 30% of patients [2-3], with 2% of cases presenting before the age of two [2]. The majority of adolescents with psoriasis experience a mild form of the disease with a roughly equal gender distribution [1], with females having an earlier onset [4]. Plaque and Guttate psoriasis have been described as the most common childhood psoriasis types [4-5], while GPP is a rare form of childhood psoriasis [3, 6-7], affecting 3% of psoriasis patients [8]. GPP is a condition with little serious chronic morbidity [7] but can be potentially life-threatening [8]. It presents abruptly with constitutional symptoms and diffuse erythematous lesions, followed by yellow-colored sterile pustules [9-10]. Patients may experience frequent flares, causing a dramatic reduction in quality of life [8]; triggers include streptococcal infections [10], emotional stress, vaccinations, and penicillin exposure [11]. While depression and a diminished quality of life have not been studied heavily in pediatric populations, the possible psychological burdens of this condition from a young age should be considered seriously [2].

In most pediatric psoriasis patients, management involves topical therapy [3, 7] and trigger avoidance [3], with the goal of reducing the number of flares [1], preserving skin surfaces, and providing physical relief from the disease [6]. Systemic therapy is reserved for patients with severe plaque psoriasis or unstable psoriatic disease, including erythrodermic psoriasis and GPP, which may progress rapidly leading to significant complications [1, 3]. Therapy for pediatric GPP is challenging [10], as there is a paucity of randomized controlled trials and standardized guidelines [1], including ones for systemic therapies [3, 9, 12]. One of the newer systemic therapies involved in treating childhood psoriasis includes biologic agents, such as etanercept, infliximab, and adalimumab [3, 9]. The objective of our study was to perform a review of the available scientific literature on biologic agents used in pediatric GPP with evaluation of therapeutic outcomes.

PubMed was utilized to identify publications that described the successful and unsuccessful use of biologic agents in pediatric pustular psoriasis. All reported cases that presented primarily as pustular psoriasis, specifically GPP, and annular pustular psoriasis (also known as subacute generalized pustular psoriasis) qualified for inclusion. Cases describing patients who presented with chronic systemic or cutaneous conditions other than pustular psoriasis did not qualify for inclusion. In studies examining multiple patients with a variety of psoriatic conditions, only pustular psoriasis cases were included in the analysis. No cases of pediatric pustular psoriasis secondary to medication use were found. Case reports, case series, and retrospective cases/case series were included. Terms searched included pediatric pustular psoriasis, pediatric generalized pustular psoriasis, childhood pustular psoriasis, pediatric annular pustular psoriasis, pediatric von Zumbusch, pustular psoriasis child, and biologics pustular psoriasis. While many biologic agents are available for clinical use, only those that have been cited in the literature for the treatment of pediatric pustular psoriasis were included in the analysis. Articles were accepted based on a review of their titles, abstracts, and full texts. If patients had more than one anti-psoriatic treatment, only those with attempted treatment with a biologic agent were analyzed, including successful biologic treatment after previously failed treatment with a differing biologic medication. Due to inconsistencies in outcome endpoints available in the present literature, response to the treatments was categorized as excellent (less than one week), good (one week to one month), or slow (> one month), and treatment outcomes were categorized as complete control, partial control, or flare during treatment. Studies examining the use of these agents in other pediatric conditions were included in the discussion due to the paucity of literature on the safety and adverse effects of biologic agents used in pediatric GPP. 

## Review

After review of the available literature, three biologic agents have been described for the treatment of pediatric generalized pustular psoriasis: etanercept, infliximab, and adalimumab. Table 1 summarizes the 12 studies identified in the PubMed search, which met inclusion criteria. 

**Table 1:** Cases of Pediatric GPP Treated with Biologic Agents *Clinical Response: Excellent when response in < 1 week; Good when response between 1 week - 1 month; Slow when response in > 1 month Abbreviations: Antibiotics: ABX, Cyclosporine: CsA, Methotrexate: MTX

Author	Failed Treatment	Medication(s) with Successful Response	Maintenance Therapy	Dose	Adverse Effects/Notes	Clinical Response*	Relapse	Treatment Outcome- Complete, Partial, or Flare during treatment (Time until Remission or last Follow-up)
Fialova [10]	Acitretin, MTX, Systemic Corticosteroids	Methylprednisolone + Etanercept + PUVA (for one recurrence)	Etanercept	0.4 mg/kg 2x/week	Candidiasis, MRSA infection	Slow	Yes	Complete (38 months)
Pereira [13]	Oral steroids, Antihistamines, ABX, Topical corticosteroids, CsA, Acitretin, Infliximab	Etanercept	Etanercept	0.4 mg/kg 2x/week for 2 months, then 0.4 mg/kg weekly	None	Good	No	Partial - residual onychodystrophy (N/A)
Mazzotta [14]	CsA, IV corticosteroids, Acitretin	Etanercept	Etanercept	0.4 mg/kg 2x/week	None	Slow	Yes	Complete (60 months)
Papoutsaki [15]	Local corticosteroids, keratolytic agents, systemic corticosteroids, Acitretin	Etanercept	Etanercept	0.4 mg/kg 2x/week for 86 weeks (12 weeks of interruption)	None	Slow	Yes	Complete (6.5 weeks)
Hawrot [16]	Topical corticosteroids, topical Tacrolimus, CsA, MTX	Etanercept + Topical corticosteroids	Etanercept (13 months)	25 mg 2x/week for 13 months	Ecchymosis at injection site	Slow	Yes	Complete (N/A)
Tsang [17]	Infliximab	Infliximab	MTX (4 months)	12.5 mg weekly, slowly tapered over 4 months	None	Excellent	No	Complete (8 months)
Skrabl-Baumgartner [18]	MTX	Infliximab	Infliximab + MTX	Infliximab 5 mg/kg at weeks 0 and 4, then every 7 weeks, plus MTX 0.3 mg/kg weekly	None	Excellent	Yes	Partial (30 months)
Glerup [19]	MTX, Anakinra	Infliximab	Infliximab	6.5 mg/kg on Day 0, 14, 28; then every 4 weeks	None	Excellent	N/A	Complete (N/A)
de Oliveira [20]	Acitretin (successful but D/C'd due to reproductive age), MTX, CsA	Infliximab	Infliximab	5 mg/kg weeks 0, 2, 6, then every 8 weeks	None	Excellent	Yes	Complete (5 months), then flare during treatment
Alvarez [22]	MTX, Acitretin, CsA, Phototherapy, Infliximab, Etanercept	Adalimumab	Adalimumab	40 mg at weeks 0, 1, then every 2 weeks	None	Slow	Yes	Flare during treatment (at 6 months), then Complete (15 months)
Callen [23]	Topical steroids, Emollients, Etretinate, UVB Phototherapy, Topical tar preparations, MTX, Etanercept, CsA, topical hydrocortisone, Isotretinoin	Adalimumab + MTX + CsA	Adalimumab	40 mg every 2 weeks	None	Slow	No	Partial - residual onychodystrophy (5 months)
Luu [21]	Topical therapy, acitretin, CsA	Infliximab Adalimumab	N/A	Infliximab: 5 mg/kg single dose Adalimumab: not specified	Infliximab successful but failed adherence to maintenance schedule	Infliximab: Excellent; Adalimumab: N/A	No	Complete (N/A)

Etanercept was a successful treatment in five patients [10, 13-16]. One patient showed a clinical response in four weeks without relapse [13], while another patient experienced a recurrence that was controlled with psoralen and ultraviolet A therapy (PUVA), in addition to the etanercept [10]. Two patients experienced an adverse effect; one patient experienced injection site ecchymosis [16], and the other patient experienced candidiasis and a MRSA infection [10], both of which were treated successfully. Aside from two recurrences of pustular lesions [10, 14] and the lack of control of onychodystrophy [13], total control was achieved in all five patients, with one publication reporting a follow-up time of 38 months [13] and another reporting a follow-up time of 72 months [14].

Infliximab was a successful treatment in five cases [17-21]. One case used infliximab as a first-line agent [17], while four others had previously failed “traditional therapies”, including methotrexate, cyclosporine, acitretin, and anakinra [18-20]. Three patients experienced total control, while one report noted remission for eight months [17] and another where the duration of remission was not included [19]. One patient experienced partial control for 30 months [18]. An 18-year-old female patient initially had successful treatment with acitretin, which was discontinued due to her childbearing age. She experienced total control with infliximab for five months until a relapse occurred. This relapse resulted in death secondary to septic shock [20]. The average time for a response to infliximab treatment in these five cases was less than one week with none of these patients experiencing adverse effects [17-21]. One patient, however, had to discontinue infliximab therapy due to an inability to attend appointments for maintenance infusions [21]. Overall, maintenance therapy was continued with methotrexate [17], infliximab [19-20], or a combination of the two [18].

Adalimumab was a successful treatment in three cases [21-23]. A 13-year-old patient, who discontinued acitretin due to liver toxicity and infliximab due to ineffectiveness in symptom reduction, thoracic pain, and acute respiratory distress, experienced a clinical response to adalimumab treatment within eight weeks. The patient experienced one relapse at 16 weeks of treatment and was treated with 40 mg of adalimumab every two weeks thereafter. She experienced a complete remission for the subsequent 15 months of follow-up. Callen, et al. described another case of pustular psoriasis successfully treated with adalimumab [23]. This 17-year old patient failed multiple therapies, including etanercept (pustular outbreak, possibly due to concurrent infection). Within two months of initiating a combination of adalimumab, methotrexate, and cyclosporine therapy, her condition was well controlled, with dramatic improvements in joint and skin symptoms. She was maintained on adalimumab monotherapy afterward, leading to the clearing of her psoriasis with minimal nail involvement [23]. Another case of successful adalimumab therapy was in an eight-year-old patient previously controlled with infliximab but failed in adherence to the maintenance infusion schedule. With adalimumab treatment, his GPP cleared and he was free of recurrences at the time of the paper [21].

Overall, GPP is a condition that can present abruptly, and research concerning the safety and efficacy of treatments varies significantly [24]. Therapy for pediatric GPP is challenging [10], as there is a lack of randomized control trials and standard guidelines [1], including those for systemic therapies [3, 9, 12]. Methotrexate, retinoids, and cyclosporine have been described as first-line agents for these patients [10, 17, 24]. In some cases, additional medications may be necessary for successful pustular psoriasis treatment [2]. While data is limited and randomized clinical trials involving the pediatric use of biologic agents are lacking, current evidence suggests these drugs may be a viable treatment option for pediatric psoriasis [2].

Biologic agents function via blocking specific inflammatory cytokines and have shown efficacy in psoriasis, a condition associated with keratinocyte hyperproliferation, and T-cell mediated inflammation [2]. The first generation of biologics targets tumor necrosis factor (TNF)-alpha, a cytokine that activates pro-inflammatory nuclear transcription factors and keratinocyte proliferation [2]. As pustular psoriasis has a cytokine profile strongly dominated by TNF-alpha [8, 25], biologics are better able to target the pathological mechanism of action [8], giving a theoretically lower risk of potential end-organ damage when compared to traditional agents [21]. In addition, due to their more convenient dosing regimens and decreased necessary serologic monitoring, biologics may be a more attractive option for the pediatric populations [21]. The first biologics developed were infliximab, etanercept, and adalimumab, all of which are approved for treating adult psoriasis [2]. TNF-alpha inhibitors, however, carry a black box warning for increased risk of malignancy, such as lymphoma, when used in pediatric patients [21].

Etanercept, a recombinant DNA-derived TNF receptor-IgG fusion protein [2, 21], has proven effective in treating adult psoriasis [2] and pediatric plaque psoriasis [15-16, 21, 26-29]. In 2008, an FDA advisory committee recommended that the FDA approve etanercept for pediatric patients with moderate-to-severe plaque psoriasis and for teens who failed alternative therapies [2]. In the European Union, etanercept has already been approved for use in refractory pediatric plaque psoriasis [21, 30-31]. Based on one study, the average time until treatment response for cutaneous psoriatic symptoms in 10 children was two to three months [32]. Siegfried, et al. examined the use of withdrawal and retreatment of moderate-to-severe pediatric plaque psoriasis and determined it to be safe for intermittent use without serious adverse side effects or infections [29]. Another study examining etanercept use in pediatric psoriasis also deemed it safe, with the most common side effect being minor infections (25% of patients) [28].

Etanercept has been shown to be well tolerated and effective in moderate-to-severe cases of plaque psoriasis [15, 26-27] through 96 weeks of treatment [26], and through 60 months of treatment in pediatric GPP [14]. Hawrot, et al. reported on nine severe recalcitrant pediatric psoriasis patients who received etanercept; seven experienced sustained improvement of their symptoms, and three had complete clearance within three months [16]. While 38% of the patients in one study experienced secondary failure of etanercept, psoriasis clearance was deemed higher with etanercept in comparison to methotrexate or cyclosporine [28].

Previous literature examining the use of etanercept in other conditions (Crohn’s disease, juvenile idiopathic arthritis (JIA), and uveitis) supports its use in pediatric patients suffering from psoriasis [30]. In 23 patients suffering from JIA, etanercept use was deemed safe to use, with patients only experiencing side effects of mild infections and no serious side effects noted [33]. Multiple publications involving JIA report no incidence of demyelination, malignancies, or death [34-35]. In addition, one JIA study found that the risk of malignancy with etanercept was not increased relative to methotrexate [36]. In addition, Lovell, et al. did not find an increase in complication rates as the length of etanercept treatment for JIA increased [35].

In contrast, however, one JIA study examining the use of etanercept found significantly more adverse side effects with etanercept use compared to methotrexate [36]. Etanercept monotherapy led to the development of incidental irritable bowel disease (IBD) and incidental uveitis, which did not occur when etanercept was used with methotrexate or with methotrexate alone [36]. Despite the uncertain relation to IBD and uveitis, they still deemed that etanercept had acceptable long-term tolerability [36]. Paller, et al. reported that three etanercept patients experienced neuropathy (non-demyelinating) [26], while some studies describe the development of sepsis (among other complications) [26, 34]. One report described that 12 of 61 pediatric JIA patients treated with etanercept withdrew from the study after developing adverse effects, all of which resolved with discontinuation of the drug [37].

The presence of such contradicting literature as it relates to the safety of etanercept supports that additional study of etanercept in the treatment of pediatric psoriasis is needed. Luu, et al. believed that among the currently available biologics, etanercept had accumulated the most evidence for efficacy and safety in the pediatric population, including pediatric psoriasis, and should be considered along with the other first-line systemic agents when treating severe or refractory psoriasis, including pustular psoriasis [21]. In cases where etanercept therapy is not successful, UVB radiation may be helpful in removing additional psoriatic lesions [38].

Infliximab is a chimeric monoclonal antibody that binds TNF-alpha, thus, preventing binding of its receptors and activating the inflammatory cascade [2, 21]. Given this mechanism of action, possible serious adverse effects include reactivation of tuberculosis and lymphoma [2]. The time of treatment onset may occur as quickly as hours to days [21]. As infliximab is part murine, it is immunogenic and results in the formation of anti-monoclonal antibody antibodies [13, 21]. Sporadic use should be avoided, as it may result in increased neutralizing antibody formation, leading to a decrease in treatment efficacy and duration, as well as transfusion reactions [13, 21].

While multiple case reports attest to infliximab’s efficacy in pediatric pustular psoriasis (see Table 1), publications studying JIA describe more adverse reactions with infliximab when compared to etanercept [21, 39-40]. Five of the 14 patients assigned to infliximab in a 2003 open-trial of infliximab and etanercept dropped out of the trial due to the development of a serious adverse effect or lack of efficacy with treatment [39]. Pontikaki, et al. described that 63% of the patients treated with infliximab experienced an adverse effect in comparison to 54.3% in the etanercept treatment group [40]. Additional immunosuppressive agents, such as methotrexate, are sometimes given to prevent the development of antibodies to infliximab [2, 18]. However, the use of infliximab as a monotherapy or concurrently with azathioprine or 6-mercaptopurine in pediatric patients has been associated with hepatosplenic T-cell lymphoma [41-42]. In addition, one case described a patient discontinuing infliximab therapy due to the failure of adherence to the maintenance infusion schedule [21]. Thus, while infliximab has proven successful in a handful of case reports for pediatric GPP, it appears etanercept may be the preferred agent [2]. One study suggests infliximab be utilized as a rescue therapy for refractory, rapidly progressive pustular psoriasis [21]. 

Adalimumab, the first fully human anti-TNF-alpha monoclonal antibody developed [2, 21], also functions by binding and preventing the interaction with TNF-alpha receptors [2]. It has been shown to have a similar safety profile to other TNF-alpha inhibitors and also includes an FDA warning about increased risk of malignancy [2]. It has proven effective in inflammatory conditions, including Crohn’s disease [43] and JIA [44-45], and has received FDA approval for use in JIA after the age of four [21, 46-47].

One study documented a higher clearance of pediatric psoriasis with adalimumab use in comparison to methotrexate and cyclosporine, with the most common side effect being minor infections (33% of patients on adalimumab) [28]. While secondary failure occurred in 33% of adalimumab patients, Garber, et al. attested to the safety of adalimumab in the treatment of pediatric psoriasis [28]. Alvarez, et al. and Callen, et al. noted that adalimumab may prove effective in patients who fail treatment with infliximab and etanercept [22-23]. As Luu, et al. stated, adalimumab is gaining popularity due to its convenient biweekly dosing and accumulating evidence of safety in the pediatric population [21]. Longer studies, however, are necessary, specifically in regards to the treatment of pediatric GPP [43].

JIA studies examining adalimumab use have also deemed it safe and effective for up to 60 weeks [33, 44-45]. When compared to methotrexate, Klotsche, et al. described an increased risk of significant adverse effects with adalimumab, but no increase in malignancies, stating that adalimumab has an acceptable long-term tolerability [36]. However, two cases of hepatosplenic T-cell lymphoma have been documented to occur with adalimumab monotherapy [48] and the prevalence of serious side effects in adalimumab treatment for JIA varies from 3.8% [45] to 78% [49], demonstrating the need for further study of this drug.

Overall, more reports on the effects and efficacy of biologic agents on pediatric psoriasis patients are slowly becoming available. If tolerated or indicated, pediatric GPP patients may begin therapy with one of the “traditional treatments”. However, if these are contraindicated, not successful, or undesired (i.e. etretinate in a reproductive-aged female), biologic agents, such as etanercept, infliximab, or adalimumab, may prove to be an effective alternative, as evidenced by the available literature. Given infliximab’s immunogenicity [13, 21] and greater adverse effect risk when compared to etanercept [21, 39-40], etanercept has been recommended as the biologic agent of choice over infliximab [2]. If etanercept monotherapy is ineffective, concurrent UVB radiation may prove helpful in removing additional psoriatic lesions [38]. Overall, the emerging presence of case reports detailing the successful use of biologic agents in pediatric pustular psoriasis adds a possible therapeutic option to “traditional therapies”. As studies examining the safety profiles of these drugs in pediatric GPP are not currently available, we must examine their safety in other pediatric conditions to gain an understanding of their possible outcomes. While these studies are informative, they do not insinuate that the demonstrated safety profiles and adverse outcomes findings translate directly to pediatric GPP patients. Every pediatric GPP patient is unique, so it is not possible to apply the safety findings described for other pediatric conditions in a vacuum. The literature on biologics use in pediatric GPP is slowly growing, and additional testing is still required to determine efficacy in a larger population, proper dosing, and adverse effect profiles [30]. 

## Conclusions

Biologics have recently gained attention in the treatment of psoriasis in pediatric patients who fail traditional therapy. While their use in adults and in pediatric plaque psoriasis is more established, their use in pediatric GPP is not well-documented. Several case reports have documented the successful use of biologic agents in refractory cases, but clinical trials are needed to gain a better understanding of effective treatment and side effect profile in this population.
